# Blinatumomab-induced lineage switch of B-ALL with t(4:11)(q21;q23) KMT2A/AFF1 into an aggressive AML: pre- and post-switch phenotypic, cytogenetic and molecular analysis

**DOI:** 10.1038/bcj.2017.89

**Published:** 2017-09-15

**Authors:** C L Haddox, A A Mangaonkar, D Chen, M Shi, R He, J L Oliveira, M R Litzow, A Al-Kali, W J Hogan, M A Elliott

**Affiliations:** 1 Department of Internal Medicine, Mayo Clinic, Rochester, MN, USA; 2 Division of Hematology/Oncology, Mayo Clinic, Rochester, MN, USA; 3 Division of Hematopathology, Mayo Clinic, Rochester, MN, USA

Lineage switch is a rare phenomenon in which acute leukemia transforms from lymphoid to myeloid lineage, or vice versa. It is typically seen following therapy or at the time of relapse.^1^ Among the chromosomal aberrations associated with lineage switch, the t(4;11)(q21;q23) rearrangement with KMT2A/AFF1 fusion protein (formerly, MLL/AFF1 or MLL/AF4) is the most common.^2^ In general, lineage switch disease is often refractory to therapy and portends a poor prognosis.^3, 4^

Blinatumomab is a monoclonal antibody with bispecificity for both CD19 on B cells and CD3 on cytotoxic T cells. Following simultaneous binding to both epitopes, normal and neoplastic B cells are lysed by the host cytotoxic T cells.^5^ Recent reports have documented lineage switch of acute leukemias following CD19-targeted therapy;^6, 7, 8, 9^ however, the underlying mechanism and management of these events are unclear. Herein, we present a case of refractory B lymphoblastic leukemia (B-ALL) with t(4:11)(q21;q23) KMT2A/AFF1 transforming to acute myeloid leukemia (AML) shortly following blinatumomab therapy. We studied both molecular and cytogenetic abnormalities at the initial diagnosis of B-ALL and at the time of lineage switch, thereby providing insights into the underlying biology.

## Case history

**Figure 1:**
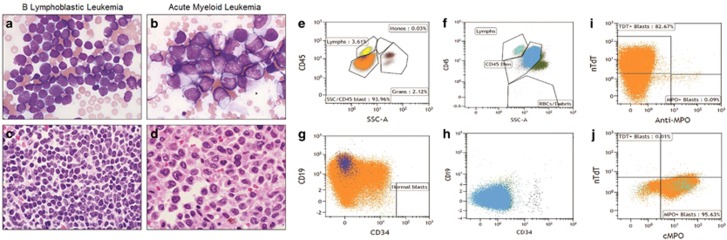
Distinct morphologic and immunophenotypic features of B-ALL blasts and AML blasts following lineage switch. (**a**) The lymphoblasts of B-ALL had scant cytoplasm, round nuclei, fine chromatin and inconspicuous nucleoli. (**b**) The myeloid blasts of AML had abundant cytoplasm, folded nuclei, fine chromatin and occasional prominent nucleoli. (**c** and **d**) Bone marrow biopsy revealed packed B-lymphoblasts with scant cytoplasm and myeloid blasts with more abundant cytoplasm, respectively. (**e, g**, and **i**) show that the lymphoblasts expressed dim CD45, lower side scatter, CD19, CD34 (partial), and terminal deoxynucleotidyl transferase (TdT). They were negative for myeloperoxidase (MPO). (**f**, **h** and **j**) show that the myeloid blasts expressed bright CD45, high side scatter and MPO, but are negative for CD19, CD34 and TdT.

A 40-year-old woman presented with vaginal bleeding. Blood work revealed a white blood cell count of 152 × 10^9^/l, anemia (hemoglobin, 9.7 g/dl) and thrombocytopenia (61 × 10^9^/l). Peripheral blood and bone marrow aspirate smears showed intermediate-sized blasts with scant, agranular cytoplasm, delicate chromatin and inconspicuous nucleoli (Figure 1a). Flow cytometric studies on peripheral blood demonstrated expression consistent with B-ALL (Table 1 and Figures 1e, g, and i). A bone marrow biopsy revealed sheets of blasts, comprising 90% of total cellularity (Figure 1c). The morphologic and immunophenotypic features were also consistent with B-ALL. Chromosome analysis revealed 46,XX,t(4;11)(q21;q23)[20] and fluorescence *in situ* hybridization (FISH) studies confirmed the AFF1/KMT2A fusion. Next-generation sequencing (NGS) of hot spot mutations of 35 genes showed a single mutation in *MPL* c.1653del; p.Lys553Argfs*77 with a variant allele frequency of 48% (Table 1). She was enrolled in a clinical trial (NCT02003222), incorporating two cycles of Berlin–Frankfurt–Münster-based induction including pegaspargase, followed by an intensification cycle with high dose methotrexate and pegaspargase. After this, patients were randomized to blinatumomab consolidation followed by further consolidation chemotherapy, versus further consolidation chemotherapy followed by planned allogeneic hematopoietic stem cell transplant. After one induction cycle, our patient had a hypocellular marrow with minimal residual disease of 5.86% measured by flow cytometry on a bone marrow aspirate, which progressed to hematologic relapse towards the end of the second induction cycle. She was removed from the clinical trial and switched to the standard HyperCVAD regimen; however, she relapsed after the first cycle. Blinatumomab salvage therapy was administered, and after 9 days, she developed leukemic infiltration of the gingiva, oral mucosa and skin. Her peripheral blood and bone marrow aspirate smears showed emergence of blasts with more abundant cytoplasm (Figure 1b). Some blasts had folded nuclei, morphologically resembling promonocytes and monoblasts. Flow cytometric studies on both peripheral blood and bone marrow aspirate showed that the blasts expressed markers consistent with AML (Table 1 and Figures 1f, h, and j). Except for the newly emerged AML blasts, there was no evidence of residual B-ALL blasts. NGS revealed persistence of the mutation in *MPL* c.1653del; p.Lys553Argfs*77 (33%) and an additional *NRAS* c.38G>A; p.Gly13Asp mutation (49%). Cytogenetic studies of the bone marrow showed a complex karyotype which contained the original t(4:11)(q21;q23). A FISH study on bone marrow aspirate confirmed the AFF1/KMT2A fusion. Table 1 summarizes the differences in abnormalities found at the time of B-ALL diagnosis and at the time of switch to AML. Subsequently, she was treated with multiple lines of salvage treatment without achieving remission. She transitioned to hospice care and passed away 208 days after her initial B-ALL diagnosis.

We present a case of refractory B-ALL harboring the t(4:11)(q21;q23) KMT2A/AFF1 fusion that rapidly transformed to AML following initiation of blinatumomab therapy. The leukemic blasts of B-ALL and AML were clonally related since they both harbored the same cytogenetic abnormality and *MPL* mutation. The complex karyotype and additional *NRAS* mutation likely represented an emerging subclone from the initial clone. The complete disappearance of the CD19-positive B-ALL blasts indicates that blinatumomab was effective in eradicating the B lymphoblasts; however, since the newly emerged AML blasts lacked CD19 expression, they escaped the cytolytic effect of blinatumomab.

While the mechanism of lineage switch remains unclear, other cases describing lineage switch after CD19-targeted therapy have postulated that clones from both myeloid and lymphoid lineages are present at diagnosis.^6, 7, 8, 9^ Following treatment, one clone may be selectively suppressed, thereby unmasking the other and manifesting as a lineage switch.^8, 9^ We re-examined the histograms of the flow cytometric studies and NGS results of the initial B-ALL blasts and failed to identify a separate AML subclone. These results suggest that a phenotypic switch took place after blinatumomab therapy. However, flow cytometry and NGS may not have sufficient sensitivity to identify a minute clone.

Other studies suggest that cell lineage plasticity mediates lineage switch in acute leukemia. In a recent report of pre-B ALL lineage switching to AML, pre-B-cell markers of single cell subclones were characterized before and after CAR-T-cell therapy. The molecular signatures of these clones varied with therapy, suggesting lineage plasticity rather than clonal selection.^8^ Experimental data in this report also demonstrated that CD19 CAR-T-cell immune pressure against CD19 can result in either relapsed leukemia with CD19 loss or leukemia with complete lineage switch as mechanisms of resistance. Using murine models, the authors demonstrated that loss of CD19 alone cannot induce lineage switch without lineage reprogramming.^8^ They suggested that a similar resistance mechanism may be seen with bi-specific CD3-CD19 antibodies like blinatumomab. In our patient, both the t(4:11)(q21;q23) rearrangement and *MPL* mutation were detected at diagnosis and after blinatumomab treatment despite the phenotypic switch, suggesting that the original clone harbored bi-lineage potential. It is possible that following blinatumomab, the cells underwent nuclear reprogramming with loss of CD19 and the adoption of a distinct myeloid phenotype.

Our case is the first to include NGS data from diagnosis and after the lineage switch. These data revealed an initial mutation in *MPL* and an additional mutation in *NRAS*. *NRAS* mutations are common in KMT2A/AFF1-positive leukemia and predict poor response to therapy and possibly therapeutic resistance.^10, 11, 12^ In our review of the literature, these mutations have not been associated with lineage switch or nuclear reprogramming previously.

While it is compelling to advocate for cautious use of blinatumomab in patients with B-ALL and t(4:11)(q21;q23) KMT2A/AFF1 rearrangements, lineage switch following CD19-targeted therapy has been observed in patients without KMT2A rearrangements.^7^ Based on our report and recent studies implicating nuclear reprogramming in the lineage switch phenomenon, characterizing the expression of nuclear reprogramming genes or changes in epigenetic memory may provide mechanistic insight into lineage switching following CD19-targeted therapy. Our case underscores the importance of thorough studies of the immunophenotypic, cytogenetic and molecular changes of the leukemic blasts during anti-CD19 therapy to promptly detect a lineage switch, which may significantly change a patient’s clinical course and therapeutic strategy.

## Figures and Tables

**Table 1:** Differences between B-lymphoblasts and myeloid blasts in flow cytometry, cytogenetics, and next generation sequencing studies at the time of diagnosis of B-ALL and post-transformation to AML 9 days following blinatumomab

	*B-ALL at diagnosis*	*AML at transformation*
Flow cytometric features of the blasts	Express: CD9, CD15 (partial), CD19, cCD22, CD38, CD45 (dim), cCD79a, HLA-DR, nTdT	Express: CD45, CD13, CD15, CD33, CD56, CD36, CD64 (partial), cMPO
	Do not express: CD10, CD3, CD13, CD16, CD33, CD117, CD2, CD7, CD56, CD36, CD64, cMPO, cCD3, CD20, CD66c	Do not express: CD34, CD19, CD10, CD3, CD16, CD117, HLA-DR, nTdT, CD2, CD7, CD38, cCD22, cCD79a, cCD3
Cytogenetics	46, XX; t(4;11)(q21;q23)[20]	52,XX,t(4;11)(q21;q23),t(5;19)(p11;q11), +6,+7,+8,+8,+13,+19[cp10]
		79-80,XXXX,-1,-2,t(4;11)(q21;q23), der(4)t(4;11),-5,-7,-9,-10,-10,-11,-15,-16, -17,-21,-22[cp9]
		45,XX,-21[1]
FISH for KMT2A-AFF1 fusion	95.60%	49%^a^
Next generation sequencing (NGS)	*MPL*: c.1653del; p.Lys553Argfs^a^77 (48%)	*MPL*: c.1653del; p.Lys553Argfs^a^77 (33%)
		*NRAS*: c.38G>A; p.Gly13Asp (49%)

Abbreviations: ALL, acute lymphoblastic leukemia; AML, acute myeloid leukemia; ‘c’, cytoplasmic; FISH, fluorescence *in situ* hybridization; ‘n’, nuclear; MPO, myeloperoxidase.

NGS genes included *ASXL1*, *BCOR*, *BRAF*, *CALR*, *CBL*, *CEBPA*, *CSF3R*, *DNMT3A*, *ETV6*, *EZH2*, *FLT3*, *GATA1*, *GATA2*, *IDH1*, *IDH2*, *JAK2*, *KIT*, *KRAS*, *MPL*, *MYD88*, *NOTCH1*, *NPM1*, *NRAS*, *PHF6*, *PTPN11*, *RUNX1*, *SETBP1*, *SF3B1*, *SRSF2*, *TERT*, *TET2*, *TP53*, *U2AF1*, *WT1* and *ZRSR2*.

a FISH was done on a follow-up bone marrow aspirate after patient was initiated on a salvage therapy for AML.
